# Single-Electron Occupation in Quantum Dot Arrays at Selectable Plunger Gate Voltage

**DOI:** 10.1021/acs.nanolett.3c03349

**Published:** 2023-12-13

**Authors:** Marcel Meyer, Corentin Déprez, Ilja N. Meijer, Florian K. Unseld, Saurabh Karwal, Amir Sammak, Giordano Scappucci, Lieven M. K. Vandersypen, Menno Veldhorst

**Affiliations:** † QuTech and Kavli Institute of Nanoscience, Delft University of Technology, PO Box 5046, 2600 GA Delft, The Netherlands; ‡ QuTech and Netherlands Organisation for Applied Scientific Research (TNO), PO Box 155, 2600 AD Delft, The Netherlands

**Keywords:** Quantum Dot, Single-electron Occupation, Uniformity, Stress Voltage, Spin Qubit

## Abstract

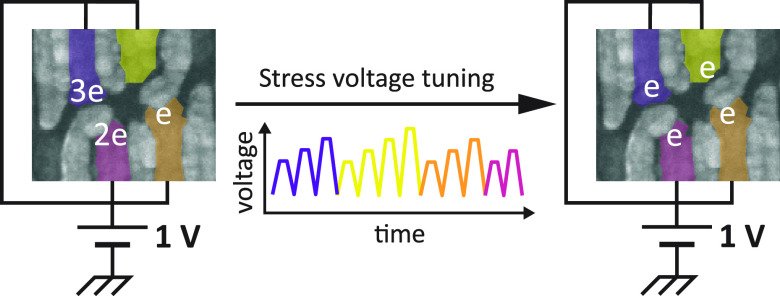

The small footprint of semiconductor qubits is favorable for scalable quantum computing. However, their size also makes them sensitive to their local environment and variations in the gate structure. Currently, each device requires tailored gate voltages to confine a single charge per quantum dot, clearly challenging scalability. Here, we tune these gate voltages and equalize them solely through the temporary application of stress voltages. In a double quantum dot, we reach a stable (1,1) charge state at identical and predetermined plunger gate voltage and for various interdot couplings. Applying our findings, we tune a 2 × 2 quadruple quantum dot such that the (1,1,1,1) charge state is reached when all plunger gates are set to 1 V. The ability to define required gate voltages may relax requirements on control electronics and operations for spin qubit devices, providing means to advance quantum hardware.

Semiconductor spin qubits have become a compelling platform for quantum computation. Single qubit gate fidelities of 99.99%^1^ and two-qubit gate fidelities exceeding 99%^2−5^ have been demonstrated. A moderate sensitivity to thermal effects allowed for the implementation of quantum operations above one Kelvin.^6−8^ Furthermore, the small size of semiconductor spin qubits and their compatibility with advanced semiconductor manufacturing^9−11^ may facilitate devices with large numbers of qubits as required for practical applications. Recent advances in the material platforms supported the realization of a 2 × 2 qubit array in germanium,^12^ a linear six qubit system in silicon,^13^ and the operation of a 16 quantum dot crossbar array.^14^ However, scaling up the number of qubits is challenging, especially when considering the numbers needed for fault-tolerant quantum computation.^15−17^ A particular challenge lies in the sensitivity of qubits to their environment leading to considerable variations of their properties, a notion that was already highlighted in the seminal work on quantum computation by Loss and DiVincenzo.^18^

Substantial reductions in variability have been achieved through progress in heterostructure growth and device fabrication. For instance, these efforts focus on reducing material disorder,^19−26^ advancing device fabrication,^27−29^ and addressing fluctuations in mechanical stress induced by the deposition of metallic gate electrodes.^30−32^ However, significant variations remain observable in current devices,^14,33,34^ and it is an open question whether sufficient uniformity can be reached through material development alone.

Alternatively, fluctuations in the potential landscape can be compensated by temporarily applying stress voltages.^35−38^ An alternating sequence of stress voltages and pinch-off measurements has already enabled on-demand reshaping of pinch-off voltage characteristics and their homogenization without signs of reduced device stability afterward. Furthermore, such sequences allow the alteration of the potential offset of a single-electron transistor (SET) at a temperature of ≈4.2 K.^38^ However, this methodology has not been applied to individual electrons in a quantum dot. Also, overcoming qubit variations in quantum processors will require the tuning of multiple quantum dots.

Here, we demonstrate the use of stress voltages to tune the potential landscape in a quantum dot array. We show that this approach allows for the change and equalization of the plunger gate voltages required to reach single-electron occupation in a double quantum dot without changing any other gate voltages. Importantly, we find that the resulting confining potential remains stable for hours afterward. To illustrate its robustness and versatility, we demonstrate that the method employed can be applied at various barrier voltages and, thus, interdot tunnel couplings. Furthermore, we show that the procedure can be extended to homogenize the plunger gate voltages defining the single occupation charge state in a 2 × 2 quantum dot system.

Figure 1a shows a scanning electron micrograph of a device nominally identical to the one under study in this work, which is fabricated on a ^28^Si/SiGe heterostructure^40^ (see Supporting Information Section S1). The gate design allows for the formation of a 2 × 2 quantum dot array (white circles) and two adjacent SETs on the left and right side.^41^ We form quantum dots Q3 and Q4 underneath plunger gates P3 and P4 and also tune the SET below sensor gate S1. The left side of the device is operated as an electron reservoir. Figure 1b depicts a charge stability diagram recorded after the initial tuning. It shows the typical honeycomb pattern of a double quantum dot and depletion down to the (*N*_3_, *N*_4_) = (1, 1) charge state with *N*_*i*_ being the charge occupation of Q*i*.

**Figure 1:**
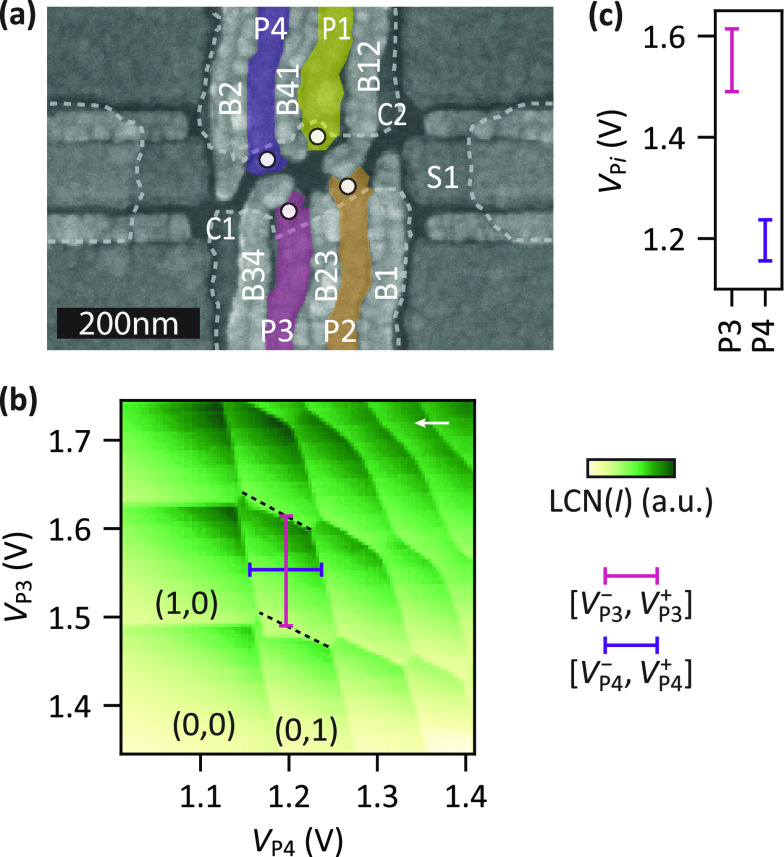
Device and tuning of a double quantum dot. (a) Scanning electron micrograph of a device nominally identical to the one under study. Confinement (C*i*) and barrier (B*i* and B*ij*) gates are designed to define four quantum dots indicated by the white circles. Their charge occupation is controlled by four plunger (P*i*) gates. Confinement gates are outlined by dashed lines for clarity. A sensor quantum dot is formed under S1 and measured in transport. (b) Charge stability diagram showing the single-electron occupation of the Q3–Q4 double quantum dot formed underneath P3 and P4. The plotted signal is locally contrast normalized (LCN) to increase the visibility of the charge transition lines as described in Supporting Information Section S1. The white arrow marks the sweep direction. Dashed lines connect charge triple degeneracy points and thereby indicate transitions of the charge ground state. These cannot be observed directly as electrons are unloaded from Q3 via Q4 leading to a dragging of charge transition lines in sweep direction (charge latching).^39^ The plunger gate voltage ranges [*V*_P*i*_^-^, *V*_P*i*_^+^] that set a (1, 1) charge state are indicated by vertical and horizontal bars. The ranges are extracted around the center point of the (1, 1) charge region (see Supporting Information Section S1). Unprocessed data shown in Supporting Information Section S8. (c) Plunger gate voltage ranges [*V*_P*i*_^-^, *V*_P*i*_^+^] as extracted in (b).

The charge stability diagram reveals a large asymmetry in the plunger gate voltages required to reach the single-electron regime. The voltage ranges [*V*_P*i*_^-^, *V*_P*i*_^+^] from the first to the second charge transition line of the two quantum dots are indicated by a horizontal and a vertical bar (see Supporting Information Section S1 for the definition). As illustrated in Figure 1c, those ranges do not overlap for the two quantum dots, and in particular, we find a separation of more than 2(4) times the Q3(Q4) charging voltage *V*_P*i*_^C^ = *V*_P*i*_^+^ – *V*_P*i*_^-^. While this is a rather extreme case, variations in the plunger gate voltages that load a single electron larger than the corresponding charging voltages are commonly observed.^14,33,42−44^ For instance, in ref (14), a variability of the first charge addition voltages of 290 mV is reported while the average charging voltage is 51 mV. Therefore, if single-electron occupation can be achieved at equal plunger gate voltages in the device of Figure 1, this would provide good prospects for the homogenization of the required plunger gate voltages, also in devices that already are intrinsically more uniform.

To increase the potential uniformity, we follow our previous work^38^ and apply stress voltages *V*_stress_ on gate electrodes to reshape the background potential landscape. We aim to tune the system such that the (1, 1) charge state is reached at a predetermined plunger gate voltage. Specifically, we target to load a single electron per quantum dot for *V*_P3_ = *V*_P4_ = *V*^T^ with *V*^T^ = 1, 1.1, and 1.2 V by sequentially tuning the potential below the two plunger gates following the path shown in Figure 2b. Figure 2a illustrates the employed procedure for a single plunger gate P*i*. We apply a stress voltage *V*_stress_ for *t*_stress_ = 1 min. Afterward, we measure charge stability diagrams around *V*_P*i*_ = *V*^T^, and if necessary, the sensor gate voltage *V*_S1_ is compensated to restore maximum sensitivity of the SET. From the charge stability diagrams, we then extract the voltage range [*V*_P*i*_^-^, *V*_P*i*_^+^] required to reach single charge occupation. If setting the target voltage does not yield the targeted electron occupation in Q*i* (*V*^T^ not in [*V*_P*i*_^-^, *V*_P*i*_^+^]), the sequence is repeated with an increased (decreased) stress voltage to shift the voltage range further upward (downward). If a single electron is loaded at the target voltage configuration, we stop applying stress voltages to P*i* and analogously tune the potential of the other quantum dot. After the initial tune up (Figure 1), we first follow the stressing procedure to lower the required plunger gate voltage ranges [*V*_P*i*_^-^, *V*_P*i*_^+^] to reach single-electron occupancy at 1 V. During this process, we adjust the barrier gate B2 voltage in order to maintain a significant tunnel rate. Then, we perform the stressing experiment and advance from point A to point E in Figure 2b. Here, we only change the sensor gate S1 voltage and keep all other gate voltages constant (see Supporting Information Section S10 for the voltage settings).

**Figure 2:**
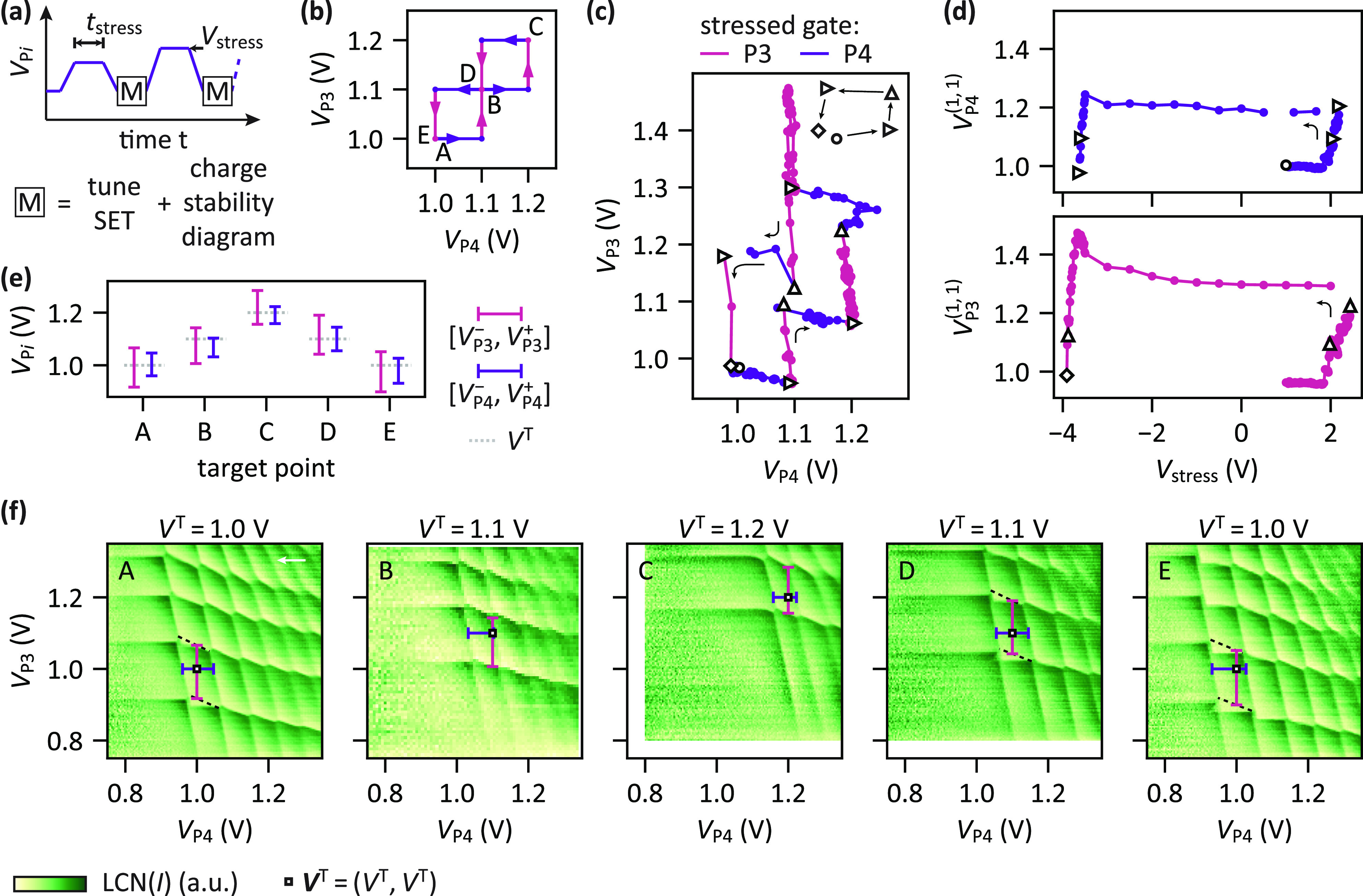
Single-electron occupation at predetermined plunger gate voltages through voltage stressing. (a) Schematic of the stress–measure sequence applied to shift the voltages required to obtain the (1, 1) charge state. Increasing stress voltages *V*_stress_ are applied for *t*_stress_ = 1 min interleaved by charge stability diagram measurements. (b) Expected trajectory for the center of the (1, 1) charge region ***V***^(1,1)^ in the (*V*_P3_, *V*_P4_) plane during the tuning procedure as defined prior to conducting the experiment. The color of the path refers to the plunger gate being stressed. (c) Actual trajectory of ***V***^(1,1)^ followed during the tuning procedure. The triangle, circles, and diamond mark the starting point, (intermediate) targets, and end point of the path, respectively. After each intermediate target, a new sequence is started as visualized by a new trace. The trace is also interrupted when insufficient contrast does not allow for obtaining ***V***^(1,1)^. Black arrows indicate the time flow. (d) *V*_P3_^(1,1)^ (bottom) and *V*_P4_^(1,1)^ (top) as a function of the applied stress voltage *V*_stress_. The triangle, circles, and diamond mark the same points as in (c), and black arrows indicate the time flow. (e) Plunger gate voltage ranges [*V*_P*i*_^-^, *V*_P*i*_^+^] that keep the double quantum dot in the (1, 1) charge state after tuning (see Supporting Information Section S1). Targets are indicated by the dotted lines. (f) Corresponding charge stability diagrams recorded after the application of the respective stress voltage sequences. The white square markers show the target voltages ***V***^T^ = (*V*^T^, *V*^T^). Plunger gate voltage ranges [*V*_P*i*_^-^, *V*_P*i*_^+^] that keep the system in the (1, 1) charge state are indicated by vertical and horizontal bars. Dashed lines indicate transitions of the charge ground state which cannot be observed directly due to a slow dot–reservoir tunneling time of Q3 (charge latching, see Supporting Information Section S1). The white arrow marks the sweep direction which is identical for all panels. Unprocessed data shown in Supporting Information Section S8.

Figure 2f shows charge stability diagrams recorded after tuning toward the predefined targets *V*^T^. A clear shift of the (1, 1) charge region to higher plunger gate voltages and then back down is observable. Furthermore, after the completion of each tuning, setting the plunger gate voltages (*V*_P3_, *V*_P4_) to ***V***^T^ = (*V*^T^, *V*^T^) (white square marker) loads a single electron per quantum dot as also highlighted in Figure 2e showing the extracted voltage ranges [*V*_P*i*_^-^, *V*_P*i*_^+^]. This demonstrates tunability of the chemical potentials and control over the electron occupation in a double quantum dot through the temporary application of the stress voltage. Due to charge latching,^39^ for lower values of *V*^T^ some charge transition lines of Q3 get dragged to the left. This suggests a crosstalk effect of the applied stress voltages on the surrounding tunnel barrier potentials.

Figure 2c shows the reconstructed evolution of the center point of the (1, 1) charge region ***V***^(1,1)^ = (*V*_P3_^(1,1)^, *V*_P4_^(1,1)^) during the tuning procedure (see Supporting Information Section S1). Overall, the experimental trajectory qualitatively reproduces the intended one shown in Figure 2b. The predominantly horizontal and vertical progressions in the (*V*_P3_^(1,1)^, *V*_P4_^(1,1)^) plane suggest limited crosstalk; i.e., applying stress voltages to one gate P*i* only has a small effect on the charge transition voltages of the quantum dot below the other plunger gate. Quantitatively, we find slopes d*V*_P*i*_^(1,1)^/d*V*_P*j*_^(1,1)^ between −0.31 V/V and −0.04 V/V. The sign of these slopes is consistent with the sign of the capacitive shift of the transition line voltage of Q*j* when the plunger gate voltage *V*_P*i*_ is changed (see Supporting Information Section S2). Correcting for this effect, we obtain the change in the charge transition voltages of Q*j* induced exclusively by the application of stress voltages set to P*i*. We find crosstalks of (+0.37 ± 0.03) V/V and (+0.19 ± 0.03) V/V for P3 on Q4 and P4 on Q3, respectively. Overall, while these crosstalk effects could be compensated for, the simple approach presented here allowed tuning of the potentials of the quantum dots to the predetermined targets.

In Figure 2d, the center voltages *V*_3_^(1,1)^ and *V*_4_^(1,1)^ are plotted as a function of applied stress voltage *V*_stress_. We recover the typical hysteresis cycle observed when tuning pinch-off voltages using an analogous method in similar devices.^38^ Noticeably, for steadily decreasing stress voltages there is an initial increase in *V*_P*i*_^(1,1)^ before it rapidly drops to lower voltages at *V*_stress_ ≈ – 4 V. In Figure 2c, this manifests as nonmonotonic progressions of ***V***^(1,1)^ between the target points C and D. *V*_P4_^(1,1)^ and *V*_P3_^(1,1)^ initially increase by 40 and 180 mV, respectively, before they decrease and approach *V*^T^ = 1.1 V.

Summarizing, Figure 2 demonstrates that the background potential in the quantum well can be reshaped such that each quantum dot can be occupied with one electron using uniform plunger gate voltages.

To understand the impact of stress voltages on device stability, we record multiple charge stability diagrams as a function of time after the initial stress tuning toward *V*^T^ = 1 V (A in Figure 2d). Figure 3a shows the extracted evolution of the plunger gate voltage range that keeps quantum dots Q3 and Q4 in single-electron occupation. Here, the time *t* refers to the time since the last application of a stress voltage and voltages are plotted relative to *V*^T^. We find that the double quantum dot system remains in the (1, 1) charge state for more than 15 h, showing only a weak drift. This is confirmed by standard deviations of 3, 3, 2, and 1 mV for *V*_P3_^-^, *V*_P3_^+^, *V*_P4_^-^, and *V*_P4_^+^, respectively, which remain negligible compared to the charging voltages of 148 and 87 mV for Q3 and Q4, respectively. Overlaying the charge stability diagrams recorded at *t* = 0 and 17 h, as depicted in Figure 3b, provides further confirmation of the device stability. Additional time traces demonstrating stability up to 40 h after the application of the last stress voltages are presented in Supporting Information Section S3. Moreover, we find that charge noise values sensed by the right SET are comparable to values typically observed in devices based on Si/SiGe (see Supporting Information Section S4 for details).

**Figure 3:**
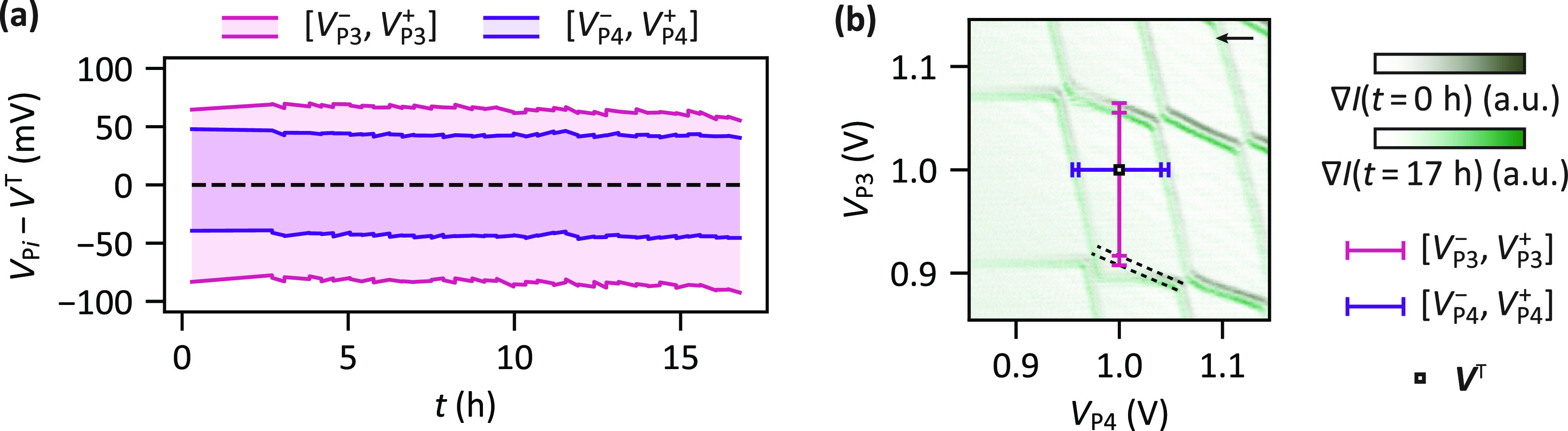
Stability of the (1, 1) charge state after stress tuning. (a) Time traces of the plunger gate voltage ranges that keep the system in the (1, 1) charge state (see Supporting Information Section S1 for the definition) after the application of a sequence of increasing stress voltages. *t* is the time after the application of the last stress voltage. Note that the underlying charge stability diagram measurements were interleaved with charge noise measurements on the sensor (see Supporting Information Section S4). Additional traces are presented in Supporting Information Section S3. (b) Overlay of charge stability diagrams taken at the beginning (olive green) and end (light green) of the time trace shown in (a). Horizontal and vertical bars indicate the respective plunger gate voltage ranges that keep the system in the (1, 1) charge state. Dashed lines indicate transitions of the charge ground state which cannot be observed directly due to a slow dot–reservoir tunneling time of Q3 (charge latching, see Supporting Information Section S1). The black arrow marks the sweep direction. Unprocessed data shown in Supporting Information Section S8.

We now address the question of whether single-electron occupation can still be achieved by a predetermined gate voltage when changing the coupling between the quantum dots. In our double quantum dot system, we can control the interdot coupling by adjusting the barrier gate B34 voltage to tune the system from strong to weak coupling quantum dots. We achieve this by varying the barrier gate voltages between 0 V and −0.5 V. After setting a barrier gate voltage, we apply stress voltages to the plunger gates to obtain the (1,1) charge state at ***V***^T^ = (1 V, 1 V). Figure 4a–e shows the resulting charge stability diagrams. Note that we do not utilize virtual gates to allow for an eased identification of the stress voltage effect. The charge transition line pattern changes from exhibiting nearly diagonal lines at *V*_B34_ = 0 mV toward a rectangular grid-like pattern at *V*_B34_ = −500 mV, revealing the transition from high to low coupling. In all cases the application of stress voltage sequences allows us to obtain the (1, 1) charge state at ***V***^T^ = (1 V, 1 V). This is confirmed by the extracted voltage ranges [*V*_P*i*_^-^, *V*_P*i*_^+^] plotted in Figure 4f. We conclude that, for a wide range of interdot couplings, single-electron occupation can be achieved at predetermined plunger gate voltage independently of the applied barrier voltage.

**Figure 4:**
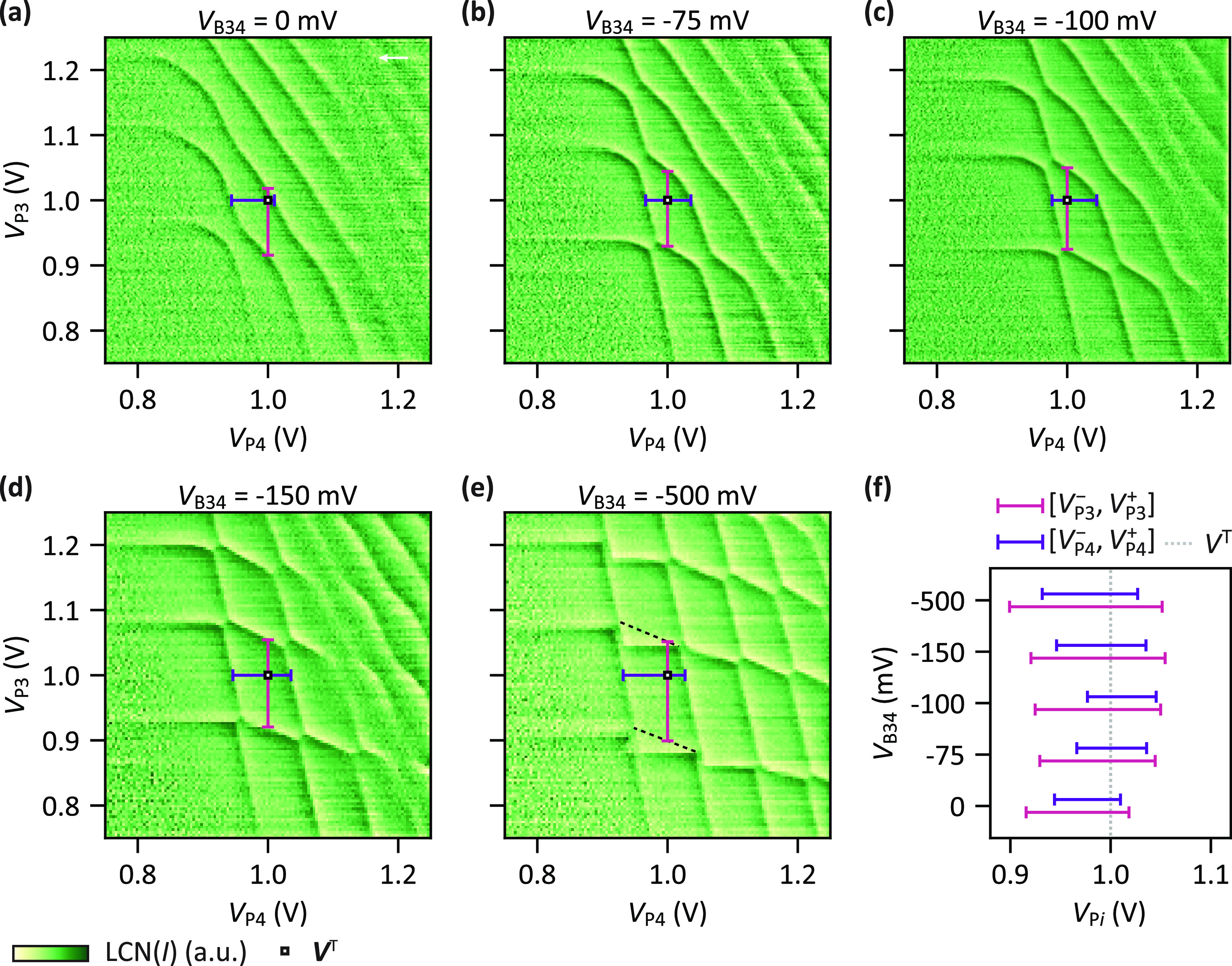
Single-electron occupation at predetermined plunger gate voltage for high and low interdot coupling. (a)–(e) Charge stability diagrams measured after tuning the system through applying stress voltages such that the (1, 1) charge state is the ground state when applying the plunger gate voltages ***V***^T^ = (1 V, 1 V) (white square marker). In each case a different barrier gate voltage *V*_B34_ is set before the tuning (labeled in the plot titles). The range of plunger gate voltages [*V*_P*i*_^-^, *V*_P*i*_^+^] that keep the system in the (1, 1) charge state is indicated by horizontal and vertical bars (see Supporting Information Section S1). Dashed lines indicate transitions of the charge ground state which cannot be observed directly due to a slow dot–reservoir tunneling time of Q3 (charge latching, see Supporting Information Section S1). The white arrow marks the sweep direction which is identical for all panels. The unprocessed data is shown in Supporting Information Section S8. (f) Plunger gate voltage ranges [*V*_P*i*_^–^, *V*_P*i*_^+^] extracted from (a)–(e). The dotted line indicates the target voltage *V*^T^ = 1 V.

Finally, we utilize our findings to tune a 2 × 2 quantum dot array such that the (*N*_1_, *N*_2_, *N*_3_, *N*_4_) = (1, 1, 1, 1) charge state is the ground state when all plunger gate voltages are set to 1 V. Starting from the Q3–Q4 double quantum dot, we form the quantum dots Q1 and Q2 which are predominantly controlled by the plunger gates P1 and P2. Then, the system is tuned solely through tailored stress voltage sequences applied to the plunger gates. Figure 5 shows two charge stability diagrams recorded after this tuning process, unveiling four sets of charge transition lines. These can be associated with the four quantum dots by analyzing further charge stability diagrams recorded by sweeping additional plunger gate combinations (see Supporting Information Section S7). Yellow, orange, red, and purple dashed lines mark the first two charge addition voltages of quantum dots Q1, Q2, Q3, and Q4, respectively. The target voltage configuration ***V***^T^ = (*V*_P1_^T^, *V*_P2_^T^, *V*_P3_^T^, *V*_P4_^T^) = (1 V, 1 V, 1 V, 1 V) is shown by a white square marker and the voltage ranges that keep the system in the (1,1,1,1) charge state are indicated by horizontal and vertical bars. ***V***^T^ clearly falls between the first two charge transition lines for all four quantum dots, confirming that we reached the targeted configuration. Here, *V*^T^ = 1 V was arbitrarily chosen, but we anticipate that other target voltages can be reached as long as the crosstalk on the interdot and dot–reservoir tunnel coupling remains negligible or is compensated for. Note that all quantum dots are strongly affected by plunger gates P2 and P4 as observable in Figure 5b. However, in Figure 5a, the voltages on P1 and P3 seem to affect only the charge occupation of Q1 and Q3. We speculate this behavior to originate from asymmetries in the gate layout and device imperfections.^41^ Crucially, we find that the stressing procedure is effective for the tuning of a nonlinear quadruple quantum dot array.

**Figure 5:**
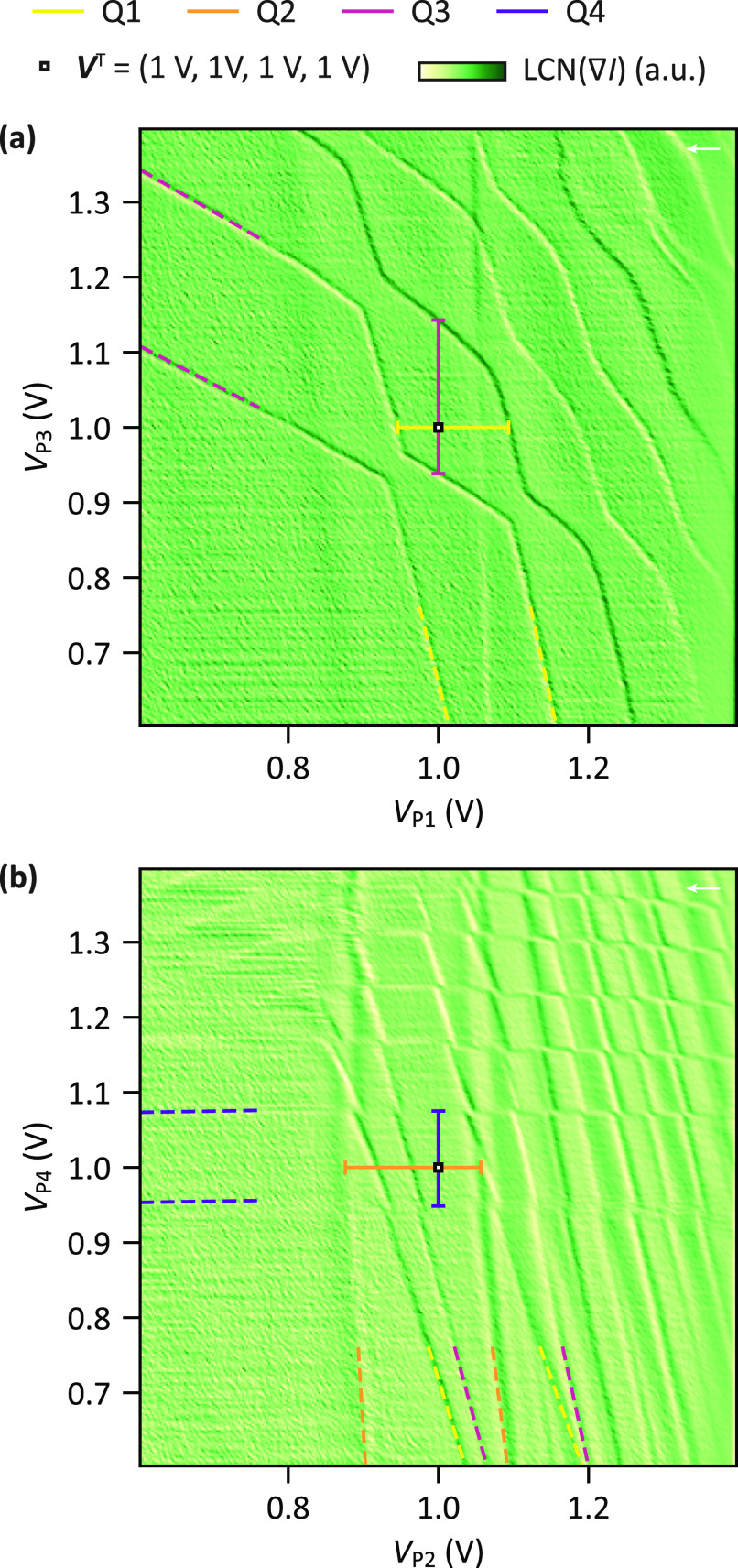
(1, 1, 1, 1) charge state at 1 V on all plunger gates (a). (b) Charge stability diagrams recorded after applying stress voltage sequences to tune the (1, 1, 1, 1) charge state to be the ground state when all plunger gate voltages are set to 1 V. The first two transition lines of each quantum dot are indicated by dashed lines. The voltage ranges to keep the system in the (1, 1, 1, 1) charge state are indicated by horizontal and vertical bars (see Supporting Information Section S1). A white square marks the point when all plunger gates are at 1 V. The plotted signal is the summation of several charge stability diagrams with identical voltage ranges recorded for slightly varied voltages on the SET plunger S1 (see Supporting Information Section S9). Contrast is enhanced by a local contrast normalization (LCN). (a) Charge transition lines of Q1 and Q3 and (b) charge transition lines of all four dots. Note that in (a) two additional vertical transition lines are present, presumably corresponding to spurious quantum dots which however show negligible coupling to Q1–Q4. The white arrows mark the sweep direction.

In summary, we have shown that single-electron occupation in quantum dots can be achieved at equal predetermined plunger gate voltage by making use of a stress-voltage based procedure. Importantly, we find that after such a tuning the systems remains stable for hours, only exhibiting small progressive drifts which do not affect the charge configuration. While our experiments suggest tunability of the entire potential landscape, more research is needed to understand the level of control over the barrier potentials. We envision that the stressing methodology may find several applications in semiconductor quantum technology. For instance, it may facilitate individual control over quantum dot potentials in crossbar arrays which crucially rely on shared gate voltages.^14,45^ Tailored stress voltages could be applied to selected gate electrodes simultaneously. The stress voltages would be chosen to leave the background potential underneath each individual gate unaffected. However, where the selected gates are in close vicinity to each other, the combined electric field would be strong enough to shift the background potential (see Supporting Information Section S5). A predetermined gate voltage to set a given charge state may also relax the requirements for control electronics and facilitate their integration. For instance, lowering the required gate voltages would allow for smaller capacitors in floating gate architectures while keeping the same refresh rate.^46^ Furthermore, we envision that stressing voltages can provide the tunability of other parameters. For example, the *g*-tensor of germanium qubits is strongly dependent on the electric field,^28,47^ such that stressing voltages may provide tunability over the qubit resonance frequency. We therefore envision that stressing procedures may become a standard and essential routine in the tuning of large quantum circuits.

## Supporting Information Available

Materials and device fabrication details, information on the experimental setup and the voltage pulses, description of the local contrast normalization, definitions of characteristic voltages, analysis of the stress voltage induced crosstalk, additional time stability time traces, charge noise characterization of the stress tuned device, proposal for local tunability in a shared gate architecture, description of potential underlying physical mechanisms, procedure to identify the four quantum dots, raw data underlying all figures, details on the processing of the data shown in Figure 5, and overview of all gate voltage configurations (PDF)

## Notes

The data and analysis supporting this work are openly available in a public Zenodo repository at 10.5281/zenodo.10254611.^48^
