# Supplementary material for: Single-Electron Occupation in Quantum Dot Arrays at Selectable Plunger Gate Voltage
Source: Nano Lett. 2023 Dec 13;23(24):11593–600. doi: 10.1021/acs.nanolett.3c03349 (PMC10755753; doi:10.1021/acs.nanolett.3c03349)
Supplement: Supplementary file 1 — nl3c03349_si_001.pdf [file nl3c03349_si_001.pdf]

Supplementary material: Single-electron occupation in quantum dot arrays at selectable plunger gate voltage

Marcel Meyer,[1] Corentin Déprez,[1] Ilja N. Meijer,[1] Florian K. Unseld,[1] Saurabh Karwal,[2] Amir Sammak,[2] Giordano Scappucci,[1] Lieven M. K. Vandersypen,[1] and Menno Veldhorst[1]

[1] *QuTech and Kavli Institute of Nanoscience, Delft University of Technology, PO Box 5046, 2600 GA Delft, The Netherlands*

[2] *QuTech and Netherlands Organisation for Applied Scientific Research (TNO), PO Box 155, 2600 AD Delft, The Netherlands*

(Dated: December 6, 2023)

CONTENTS

S1. Material and methods 1
  Heterostructure and device fabrication 1
  Setup and voltage pulses 2
  Local contrast normalization 2
  Extraction of characteristic voltages from charge stability diagrams 2

S2. Stress voltage induced crosstalk 2

S3. Additional time traces recorded after applying stress voltages 4

S4. Charge noise after applying stress voltages 4

S5. Stress voltage tuning in shared gate architectures 6

S6. Underlying physical mechanisms 7

S7. Identification of the four quantum dots 7

S8. Raw data underlying Fig. 1-4 of the main text 9

S9. Raw data underlying Fig. 5 of the main text 10

S10. Overview of applied gate voltage configurations 13

References 14

S1. MATERIAL AND METHODS

Heterostructure and device fabrication

The device under study in this work is fabricated on a ^{28}Si/SiGe heterostructure [1] which is based on a Si wafer. First, a linearly graded $Si_{1-x}Ge_x$ buffer with x varying from 0 to 0.3 is grown followed by a 300 nm relaxed $Si_{0.7}Ge_{0.3}$ layer. A 7 nm purified (800 ppm) ^{28}Si layer defines the quantum well and is separated from the gate stack by another 30 nm thick relaxed $Si_{0.7}Ge_{0.3}$ buffer that is passivated in dichlorosilane at 500 °C. Phosphorus ion implantation is utilized to contact the two dimensional electron gas and a 10 nm aluminum oxide layer precedes the deposition of gate electrodes. The latter are spread across three layers and made of Ti/Pd deposited via electron beam evaporation. They are separated by 5 nm thick layers of aluminium oxide. In all cases aluminium oxide is deposited via atomic layer deposition [2].

Setup and voltage pulses

All measurements are performed in a dilution refrigerator at a base temperature of $\approx$ 20 mK. The gate voltages are supplied by digital analog converters (DACs) with a resolution of 18 bit and a voltage range of $\pm$4 V which was amplified to $\pm$20 V for the plunger gates. The current through the SET is measured via a current-to-voltage converter connected to a digitizer module. Confinement and stress voltages are applied via the DACs while charge stability diagrams are recorded by sending fast voltage pulses. The latter are generated by an arbitrary waveform generator (AWG). DAC and AWG voltage signals are merged with a bias tee located on the sample PCB at the mixing chamber stage. AWG pulses are modified to correct for voltage drifts caused by (dis)charging of the bias tees. Furthermore, cross-capacitive shifts from P3 and P4 on the sensing dot potential are compensated for by proportionally adjusting V_{S1} when sweeping the plunger gate voltages $V_{\mathrm{P}i}$ ($\Delta V_{\mathrm{S1}}/\Delta V_{\mathrm{P}i} < 0.01$).

Local contrast normalization

In voltage scans spanning a large range, cross-capacitive coupling of the plunger gates to the SET can cause significant variations in sensor sensitivity. This leads to contrast fluctuations across the charge stability diagram and hampers identification of charge transition lines. We compensated for this effect by applying a local contrast normalization (LCN). In essence, a smoothed charge stability map is subtracted to compensate for a slowly varying offset after which a smoothed local variance is utilized to locally normalize the signal:

$$\mathrm{LCN}(I) = \frac{I - I * f_{\mathrm{Gaussian}}}{\sqrt{(I - I * f_{\mathrm{Gaussian}})^2 * f_{\mathrm{Gaussian}}}}$$

Here, the asterisk denominates a convolution, I is the sensor signal and f_{Gaussian} refers to a normal distribution with a mean and variance chosen between 4 and 50 pixels.

Extraction of characteristic voltages from charge stability diagrams

For each charge stability diagram we identify the coordinates of the charge triple degeneracy points (triple points) that constitute the corners of the (1,1) charge region. From these we calculate the voltage ranges $[V_{\mathrm{P}i}^{-}, V_{\mathrm{P}i}^{+}]$ that keep the system in the (1,1) charge state around the center point $\boldsymbol{V}^{(1,1)}$ (in Fig. 1.b of the main text) or the target voltages $\boldsymbol{V}^{\mathrm{T}}$ (in all other figures of the main text). The center point $\boldsymbol{V}^{(1,1)}$ of the (1,1) charge region is determined as the centroid of the triple points at the $(2,0)-(1,1)$ and $(1,1)-(2,0)$ charge transitions. Note that the voltage ranges $[V_{\mathrm{P}i}^{-}, V_{\mathrm{P}i}^{+}]$ are a measure of the maximum voltage variation on a single plunger gate for which the charge state remains constant. When taking into account more than a single gate voltage a polytope describes the applicable gate voltages that keep the charge state at single electron occupation. For instance, when considering two plunger gates the polytope would be the hexagon typically found in a double quantum dot honeycomb pattern. While we utilize one-dimensional voltage ranges $[V_{\mathrm{P}i}^{-}, V_{\mathrm{P}i}^{+}]$ to ease visualizations, after all stressing experiments the target voltage point $\boldsymbol{V}^{\mathrm{T}}$ lies inside the single charge occupation region (inside the respective gate voltage polytope).

We have used the triple points for the analysis because of their robustness against latching effects [3]. For instance, in Fig. 1.b of the main text, the dashed lines show reconstructed charge transition lines of quantum dot Q3 which has a weak coupling to the nearby charge reservoir. Electrons in Q3 are unloaded via Q4 when the potential of Q4 aligns with the Fermi level. Consequentially, charge transition lines are dragged in sweep direction. Therefore, $[V_{\mathrm{P}i}^{-}, V_{\mathrm{P}i}^{+}]$ can include regions of meta-stable charge state (in between the observed and the reconstructed charge transition). This does not impact our conclusions because, at the end of all stressing experiments, the target voltage point $\boldsymbol{V}^{\mathrm{T}}$ lies in a region of stable charge state.

S2. STRESS VOLTAGE INDUCED CROSSTALK

A stress voltage applied to a plunger gate Pj not only alters the potential of the quantum dot Qj located directly underneath it but also affects neighbouring quantum dots Qi. We investigate this crosstalk by further analyzing the tuning of the Q3-Q4 double quantum dot presented in Fig. 2 of the main text. Fig. S2.a shows the trajectory of the center $\boldsymbol{V}^{(1,1)}$ of the (1,1) charge state region in the $(V_{\mathrm{P3}}, V_{\mathrm{P4}})$ plane (same as Fig. 2.c of the main text). The crosstalk manifests as a deviation from perfectly horizontal or vertical progressions of $\boldsymbol{V}^{(1,1)}$. We quantify it by applying a

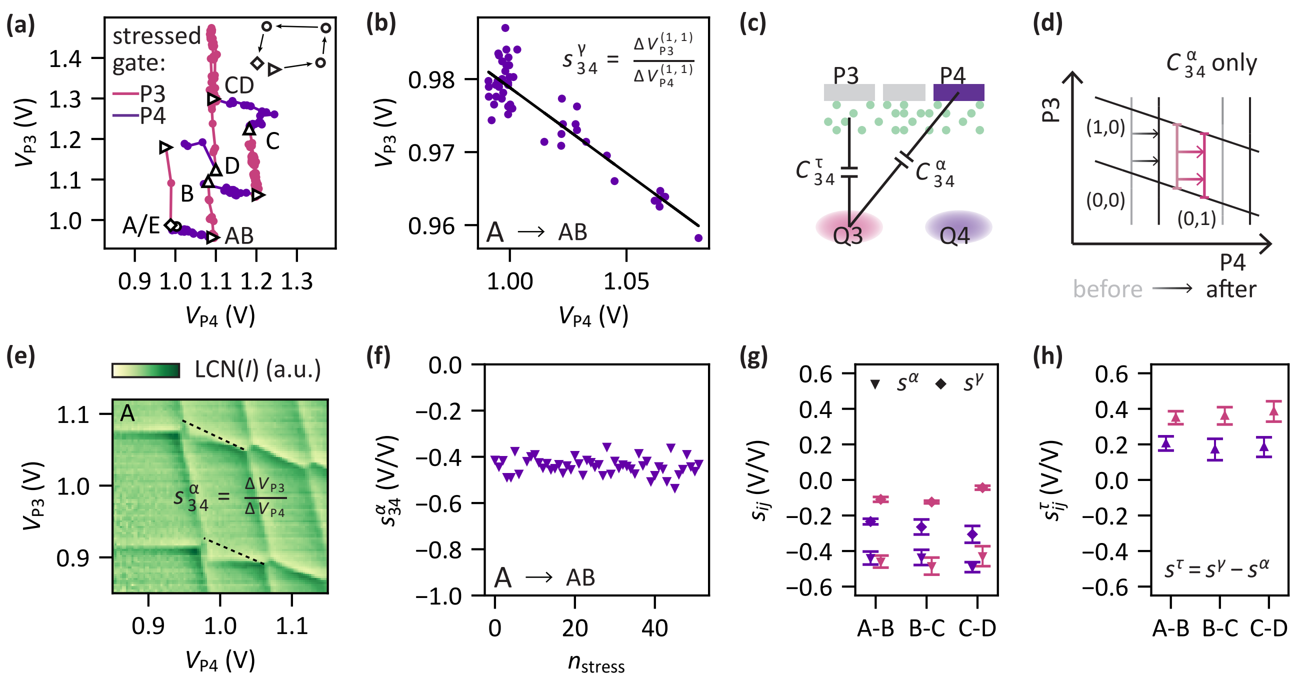

Figure S1. **Stress voltage induced crosstalk on quantum dots.** **(a)** Trajectory of the (1,1) charge state center point $\boldsymbol{V}^{(1,1)}$ in the $(V_{\text{P3}}, V_{\text{P4}})$ plane during the tuning experiment shown in Fig. 2 of the main text (identical to Fig. 2.c). **(b)** Part of the trajectory between the points A and AB. The black line is a linear fit to the data to determine the slope s^{γ}_{34} that quantifies the crosstalk of plunger gate P4 on quantum dot Q3. **(c)** Illustration of a device cross section portraying the capacitive effect of the plunger gate voltage V_{P4} on the potential of quantum dot Q3 (C^{α}_{34}) and the crosstalk effect of applying stress voltages to plunger gate P4 on the potential of quantum dot Q3 (C^{τ}_{34}). **(d)** Schematic charge stability diagram illustrating how the charge transition voltages of quantum dot Q3 shift when changing the voltage on plunger gate P4. Grey lines indicate the charge transition lines before and black lines after changing the potential of Q4 through applying stress voltages. For illustration purposes the interdot coupling between Q3 and Q4 and the capacitive coupling of P3 onto Q4 are neglected. **(e)** Example charge stability diagram taken at point A in **(a)**. The slope s^{α}_{34} of the transition lines of Q3 (black dashed lines) are determined as a measure for the relative capacitive effect of plunger gate P4 onto the potential of quantum dot Q3. To ensure robustness against distortions from charge latching effects, the Q3 charge transition lines are defined as the lines connecting the respective triple charge degeneracy points. **(f)** All extracted s^{α}_{34} during the tuning from point A to AB in **(a)**. **(g)** Crosstalk s^{γ}_{ij} caused by stressing plunger gate Pi (diamonds) and cross-capacitance effect s^{α}_{ij} of plunger gate voltage $V_{\text{P}j}$ (downward pointing triangles) onto the potential of quantum dot Qi along the trajectory in **(a)**. Between C and CD and CD and D only the last ten points are fitted to extract s^{γ}_{ij}. Due to a limited number of data points no values are shown for the tuning between D and E. **(h)** Stress voltage induced crosstalk effect s^{τ}_{ij} of plunger gate Pj onto the potential of quantum dot Qi corrected for the capacitive coupling of plunger gate Pj onto the potential of quantum dot Qi.

linear regression as exemplary shown in Fig. S2.b for the section from A to AB. The extracted slope s^{γ}_{34} is a measure for the crosstalk of plunger gate P4 onto quantum dot Q3.

Two mechanisms can explain the observed crosstalk as illustrated in Fig. S2.c: (1) Tuning the potential landscape of Q4 through the application of stress voltages also affects the potential of Q3 even if all gate voltages are reset to their initial value afterwards. For instance, this effect could be caused by the (de)charging of traps at the interface that capacitively couple to Q3 (C^{τ}_{34}) [4–8]. (2) $V^{(1,1)}_{\text{P3}}$ is defined as the middle point between the (1,0)-(1,1) and (1,1)-(1,2) charge transition at $V_{\text{P4}} = V^{(1,1)}_{\text{P4}}$ (and vice versa). Due to the capacitive coupling of P4 onto Q3 (C^{α}_{34}) a shift in $V^{(1,1)}_{\text{P4}}$ is therefore also reflected in $V^{(1,1)}_{\text{P3}}$. Fig. S2.d portrays the mechanism. It shows a schematic charge stability diagram before (grey charge transition lines) and after (black charge transition lines) tuning the potential below P4 through the application of stress voltages. As the Q3 charge transition lines are tilted by the cross-capacitance C^{α}_{34}, a change in $V^{(1,1)}_{\text{P4}}$ also results in a change of $V^{(1,1)}_{\text{P3}}$ (center point of the light and dark pink vertical bar).

To quantify the latter effect we determine the slope s^{α}_{34} of the Q3 charge transition lines at the (1,1) charge region. Fig. S2.e depicts an exemplary charge stability diagram during the tuning process with the respective Q3 charge transition lines indicated by dashed lines. All extracted s^{α}_{34} between the points A and AB in Fig. S2.a are plotted in Fig.S2.f. We find that s^{α}_{34} remains constant throughout the entire stress voltage sequence from A to AB.

The same analysis steps are repeated for all subparts between A and D of the trajectory in Fig. S2.a. Fig. S2.g summarizes all s_{ij}^{γ} (diamonds) and s_{ij}^{α} (downward pointing triangles). The magnitude of the cross-capacitance effect s_{ij}^{α} is consistently larger than the magnitude of the measured crosstalk s_{ij}^{γ}. To estimate the stress voltage crosstalk s_{ij}^{τ} solely caused by shifts of the intrinsic potential we subtract s_{ij}^{α} from s_{ij}^{γ} and plot the difference in Fig. S2.h. We find a positive voltage stress related crosstalk, which has a similar magnitude as the capacitive effect s_{ij}^{α}. As s_{ij}^{τ} and s_{ij}^{α} have a different sign they partially cancel each other and lead to a reduced effective crosstalk s_{ij}^{γ} when applying stress voltage sequences.

S3. ADDITIONAL TIME TRACES RECORDED AFTER APPLYING STRESS VOLTAGES

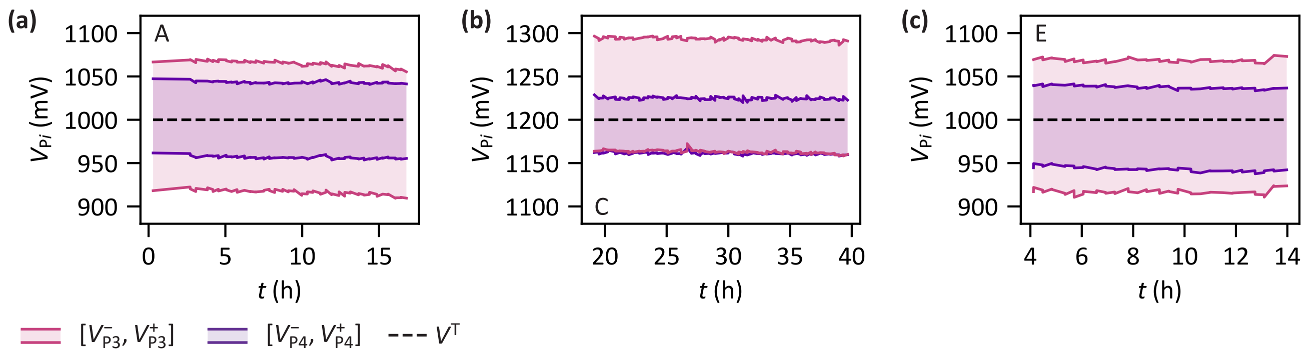

Figure S2. **Additional time traces after applying stress voltage sequences. (a)-(c)** Time traces of the voltage ranges $[V_{Pi}^{-}, V_{Pi}^{+}]$ after the application of a stress voltage sequence. **(a)**, **(b)** and **(c)** are recorded after tuning to the target points A, C and E as presented in Fig. 2 of the main text, respectively. t is the time after the application of the last stress voltage. **(a)** is identical to Fig. 3.a of main text. Note that the underlying charge stability diagram measurements were interleaved with charge noise measurements on the sensor (see supplementary section S4).

Fig. S3 shows two additional time traces not shown in Fig. 3 of the main text. Note that in Fig. S3.b and c the recording of the time traces was started 20 h and 4 h after the application of the last stress voltage, respectively. The additional curves confirm that after the application of a stress voltage tuning the system remains in a (1,1) charge state for 40 h at least only exhibiting small progressive drifts.

S4. CHARGE NOISE AFTER APPLYING STRESS VOLTAGES

As the presented tuning procedure might alter the configuration of charge traps in the heterostructure (see supplementary section S6) we investigate the system charge noise after applying stress voltages. Specifically, we measure time traces of the current through the sensing quantum dot (underneath S1) and compute the power spectral density (PSD). To obtain maximum sensitivity of the sensor current to potential fluctuations we tune the sensor plunger gate voltage V_{S1} to the flank of a Coulomb peak. Fig. S3.a, b and c depict PSD spectra obtained after tuning to the target point A, C and E in Fig. 2.b, respectively. Note that target points A and C are reached by applying positively signed stress voltages and target point E is reached by applying negatively signed stress voltages. The charge noise curves follow the typical $1/f$ frequency dependence. Therefore we fit them between 0.1 Hz and 5 Hz with $S_{\epsilon}^{\text{fit}} = A \times f^{-\kappa}$ (black line). We find noise amplitudes of $\sqrt{A} = 0.71$ μeV/Hz$^{1/2}$, $\sqrt{A} = 0.60$ μeV/Hz$^{1/2}$ and $\sqrt{A} = 0.78$ μeV/Hz$^{1/2}$ as well as exponents $\kappa = 0.96$. $\kappa = 1.38$ and $\kappa = 1.07$ for target point A, C and E, respectively. These values are comparable to charge noise amplitudes in Si/SiGe reported in the literature [9–11] and charge noise values measured in the same device during an earlier cooldown [1]. However, further research is required as the charge noise sensed by the sensor might not be representative of the charge noise affecting qubits that are tuned in the quantum dots.

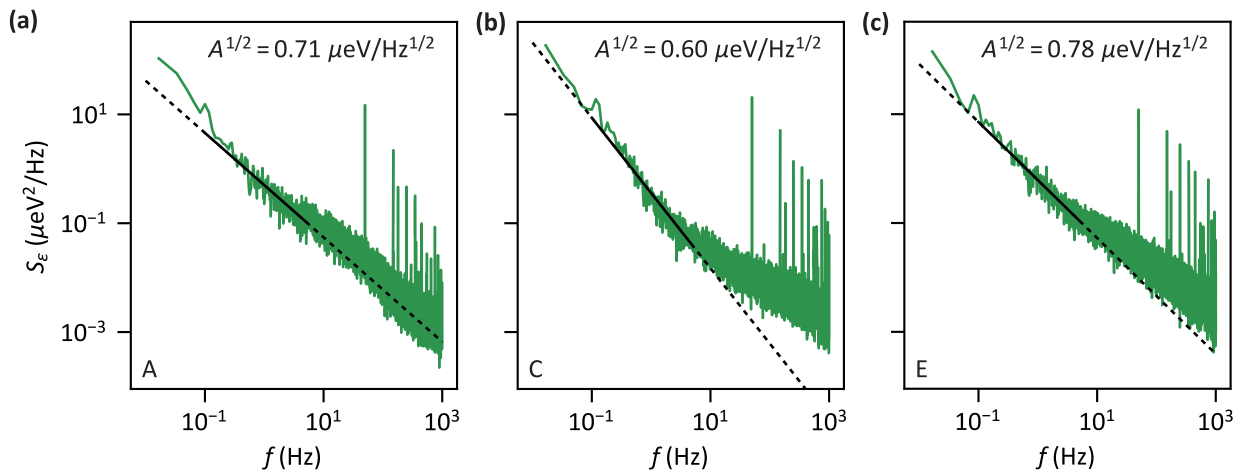

Figure S3. **Sensor charge noise after applying stress voltages.** **(a)** Power spectral density (PSD) extracted from sensor current time traces recorded after tuning to point A in Fig. 2.b of the main text. $S_\epsilon = \alpha^2 S_I/|dI/dV_{\mathrm{S1}}|$ with α the lever arm of sensor plunger gate S1 extracted from coulomb diamonds, $|dI/dV_{\mathrm{S1}}|$ the maximum slope of the coulomb peak and S_I the PSD of the current through the sensor [9]. For the measurement the sensor plunger voltage V_{S1} is tuned to the Coulomb peak flank, the voltage for which the sensing quantum dot is most sensitive to potential fluctuations. The black line is a fit to S_ϵ between 0.1 Hz and 5 Hz with $S_\epsilon^{\mathrm{fit}} = A \times f^{-\kappa}$. The noise amplitude A at 1 Hz is given in the upper right. $\kappa = 0.96$ **(b)** and **(c)** Same as **(a)** but recorded after reaching target point C and E in Fig. 2.b of the main text, respectively. $\kappa = 1.38$ for C and $\kappa = 1.07$ for E.

S5. STRESS VOLTAGE TUNING IN SHARED GATE ARCHITECTURES

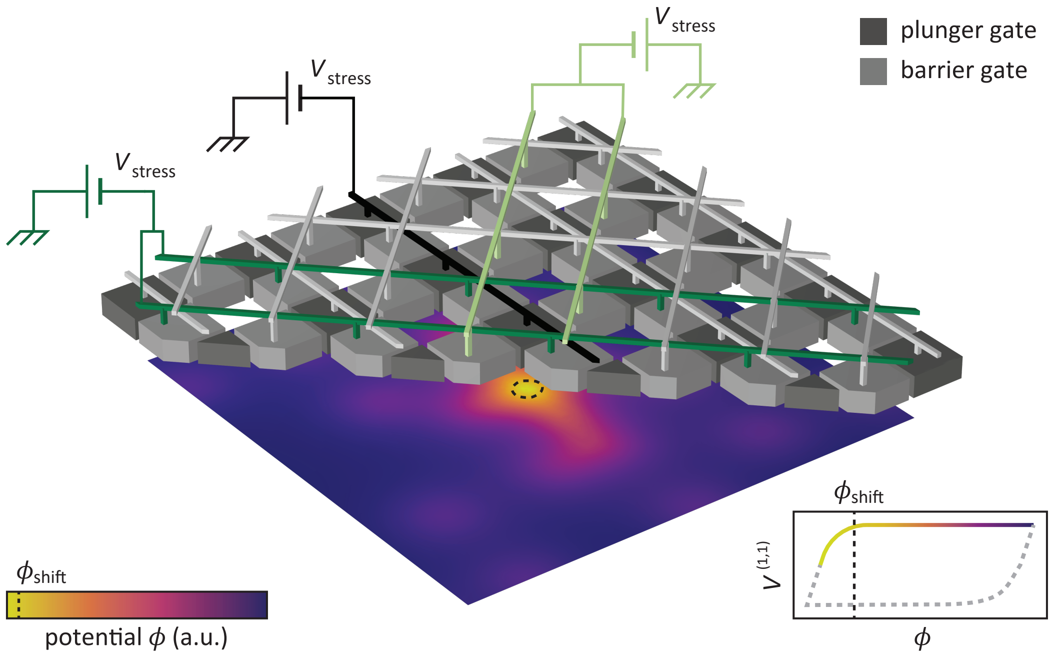

Figure S4. **Proposal for local tunability in a shared gate architecture.** A grid of gate electrodes intended to form a two-dimensional quantum dot array is shown in gray. Plunger gates are depicted in dark gray and barrier gates in light gray. Wires above the gates schematically indicate the routing of applied gate voltages. Plunger electrodes share voltages column-wise and barrier gates share voltages diagonally forming a crossbar architecture. Stress voltages $V_{\rm stress}$ are applied to a set of column wires and diagonal wires as indicated. The resulting electrical potential ϕ is illustrated below the gate electrodes. Shifting the background potential via the application of stress voltages requires that the electrical potential crosses a threshold value $\phi_{\rm shift}$ as illustrated by a schematic hysteresis curve in the bottom right (also see Fig. 2.d in the main text). $V_{\rm stress}$ is chosen such that below each stressed gate individually ϕ does not exceed $\phi_{\rm shift}$. Thus, no hysteretic shift of the background potential is induced. However, where multiple stressed gate electrodes are in close vicinity to each other (near the crossing points of the wiring), the combined electric potential exceeds $\phi_{\rm shift}$ and thus is strong enough to induce a shift in the background potential. This could enable local tunability of the electric potential, in the depicted case below the central plunger gate. A sequential application of the method to multiple combinations of gate groups may enable individual tunability of all quantum dots and tunnel barriers in a shared gate architecture.

S6. UNDERLYING PHYSICAL MECHANISMS

Applying a stress voltage to a selected gate electrode possibly alters the occupation of charge traps in the gate dielectrics and heterostructure directly underneath [4–8]. As the electric field bends the conduction band electrons might tunnel into or out of these charge traps. Removing the stress voltage then effectively freezes their occupation which permanently alters the intrinsic potential landscape. Charge traps can be present in the oxide layer [12–15], originate from unpassivated silicon and germanium dangling bonds [13–15] or arise from mechanical stress induced by the deposition of metallic gate electrodes [16, 17]. Furthermore, also the relocation of mobile ions might change the intrinsic potential [18]. Note that these processes in general are independent of the quantum well material itself and stress-voltage-controlled shifts of the intrinsic potential also have been observed in Ge/SiGe heterosturctures [19, 20].

S7. IDENTIFICATION OF THE FOUR QUANTUM DOTS

In order to identify the quantum dots visible in Fig. 5 of the main text we measure multiple charge stability diagrams by sweeping all pairwise combinations of the device plunger gate voltages. The obtained charge stability diagrams are plotted in Fig. S5. The center left and bottom center panel are identical with the charge stability diagrams shown in Fig. 5 of the main text. All maps are obtained at the same gate voltage configuration and at their center point all plunger gates are set to 1 V.

The charge stability diagrams can be analyzed starting from one charge transition line, e.g. the first vertical charge transition line in the center left panel (indicated by a yellow dashed line). Due to its strong coupling to plunger gate P1 we identify it as a charge transition line of quantum dot Q1. We mark the crossing point of this Q1 charge transition line with the $V_{\mathrm{P1}} = 1$ V line (vertical white line) by a yellow circle. Then we place another yellow circle marker at identical V_{P3} on the $V_{\mathrm{P2}} = 1$ V line in the center panel of the figure. The vertical white lines inside one row of figure panels are identical line cuts in the gate voltage space. Therefore both marked points identify the same charge transition line of the same quantum dot (Q1). Analogously two charge stability diagrams in one column of figure panels can be compared. By repeating the process for all neighbouring charge stability diagrams one can identify the charge transition lines of four quantum dots Q1-Q4. Note that the charge transition lines of quantum dot Q4 (purple) latch when the sweep direction (black arrow in the upper right of each panel) is nearly perpendicular to the charge transition lines. Therefore the crossing point of the first Q4 charge transition line with the $V_{\mathrm{P1}} = 1$ V line in the bottom left panel and the crossing point with the $V_{\mathrm{P3}} = 1$ V line in the bottom right panel differ from the crossing point with the $V_{\mathrm{P2}} = 1$ V line in the bottom center panel. Furthermore, in the left column another nearly vertical charge transition line is visible in the background. However, it shows negligible coupling to the other charge transition lines and likely is a signature of a spurious defect quantum dot outside but close to the active device region.

Additionally, table S7 shows the relative lever arms as extracted from the charge stability diagrams in Fig. S5.

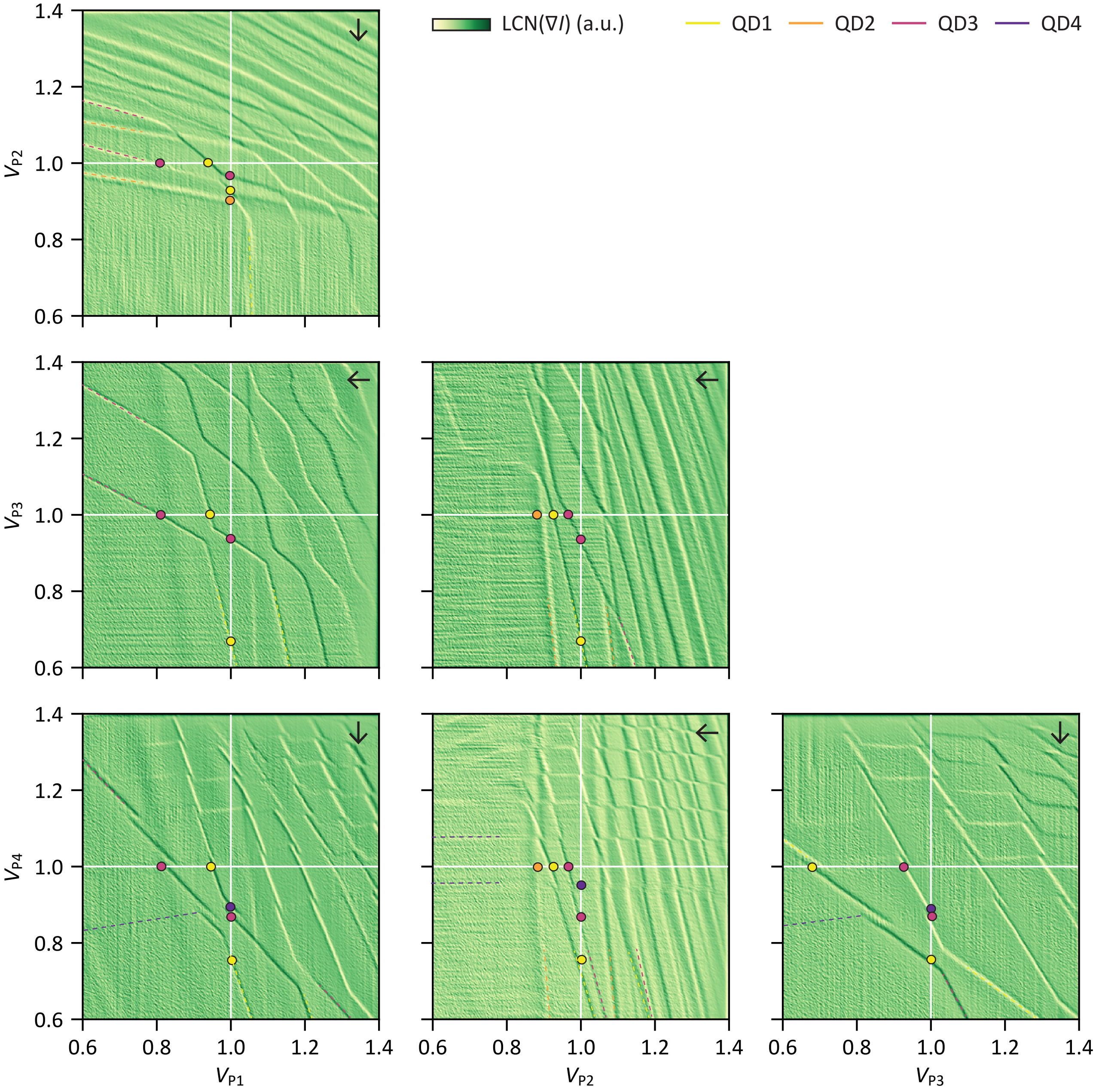

Figure S5. **Charge stability diagrams identifying the quantum dots Q1-Q4.** Charge stability diagrams recorded for all plunger gate combinations. The arrow in the upper left corner indicates the sweep direction. The center point (crossing point of the white lines) for each charge stability diagram corresponds to the same voltage configuration with $V_{P1} = V_{P2} = V_{P3} = V_{P4} = 1$ V. Horizontal white lines mark identical line cuts in the gate voltage space inside each column of charge stability diagrams. Vertical white lines mark identical line cuts in the gate voltage space inside each row of charge stability diagrams. Colored dashed lines indicate charge transitions and colored circles mark crossing points of the charge transitions with the white lines. Each color refers to a quantum dot as indicated by the legend in the upper right. To enhance the visibility of the charge transition lines the derivative of the sensor current was taken and a local contrast normalization (LCN) was applied.

	P1	P2	P3	P4
Q1	1	0.82	0.26	0.35
Q2	0.18	1	0.09	0.09
Q3	0.53	2.06	1	0.55
Q4	-	-	-	1

Table S1. **Cross coupling matrix.** The table shows the relative lever arms of the plunger gates P1-P4 to the quantum dots Q1-Q4. The displayed values are obtained by extracting transition line slopes in the charge stability diagrams of Fig. S5. The influence of plunger gate Pi on quantum dot Qj is given by the slope $\frac{\Delta V_{\mathrm{P}j}}{\Delta V_{\mathrm{P}i}}$ of the Qj transition line. Slopes are extracted for the first addition line at the lowest feasible charge occupation. The matrix diagonal is set to 1. Values for Q4 could not be obtained reliably as the Q4 transition is dragged due to charge latching.

S8. RAW DATA UNDERLYING FIG. 1-4 OF THE MAIN TEXT

Fig. S6, S7, S8, and S9 display the unprocessed charge stability diagram data underlying Fig. 1.b, 2.f, 3.b, and 4.a-e of the main text, respectively.

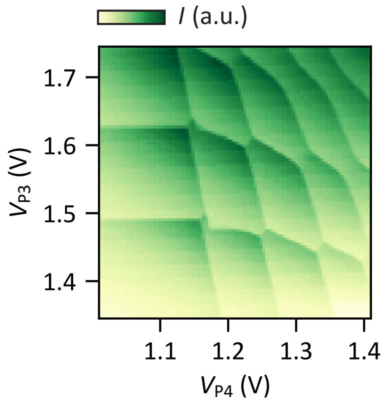

Figure S6. **Raw data underlying Fig. 1.b. of the main text**

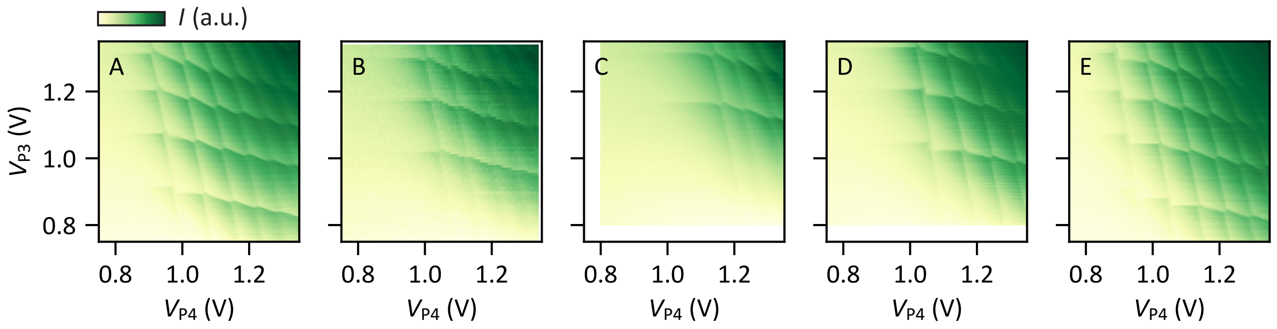

Figure S7. **Raw data underlying Fig. 2.f of the main text.**

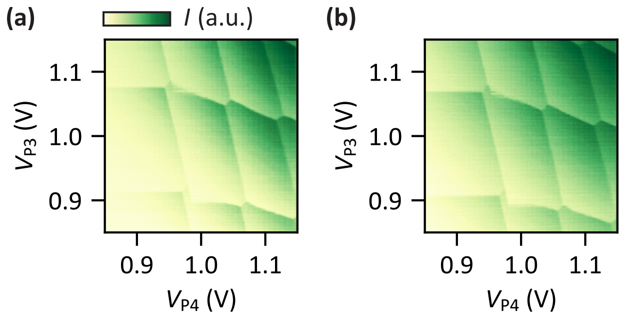

Figure S8. **Raw data underlying Fig. 3.b of the main text.** **(a)** Charge stability diagram taken at the beginning of the time trace shown in Fig. 3.a of the main text. **(b)** Charge stability diagram taken at the end of the time trace shown in Fig. 3.a of the main text.

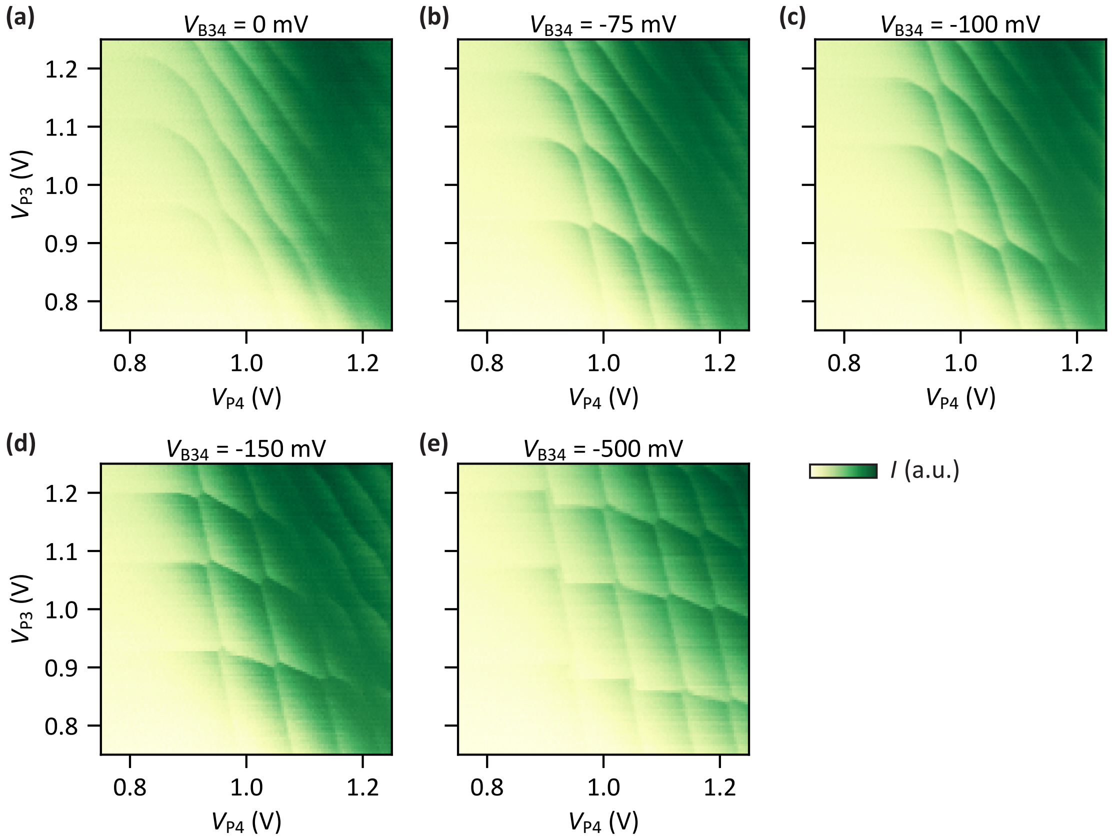

Figure S9. **Raw data underlying Fig. 4 of the main text.** **(a)-(e)** correspond to Fig. 4.a-e of the main text, respectively.

S9. RAW DATA UNDERLYING FIG. 5 OF THE MAIN TEXT

Fig. S10 and Fig. S11 show the unprocessed charge stability diagram data underlying Fig. 5.a and b of the main text, respectively. Each map is recorded at a different sensor gate S1 voltage to account for the cross-capacitance effect of the plunger gates on the sensing dot potential which limits the sensing dot sensitivity to small plunger gate voltage ranges.

We combine the charge stability diagrams by summing up the sensor current signals as exemplary shown in Fig. S12.a for the data shown in Fig. S11. Afterwards, the signal gradient ∇I is calculated as depicted in Fig. S12.b. Finally, a local contrast normalization (see methods section) is applied to allow for an eased identification of charge transition lines across the full map. Fig. S12.c depicts the resulting charge stability diagram which is identical to the charge

stability diagram shown in Fig. 5.b of the main text.

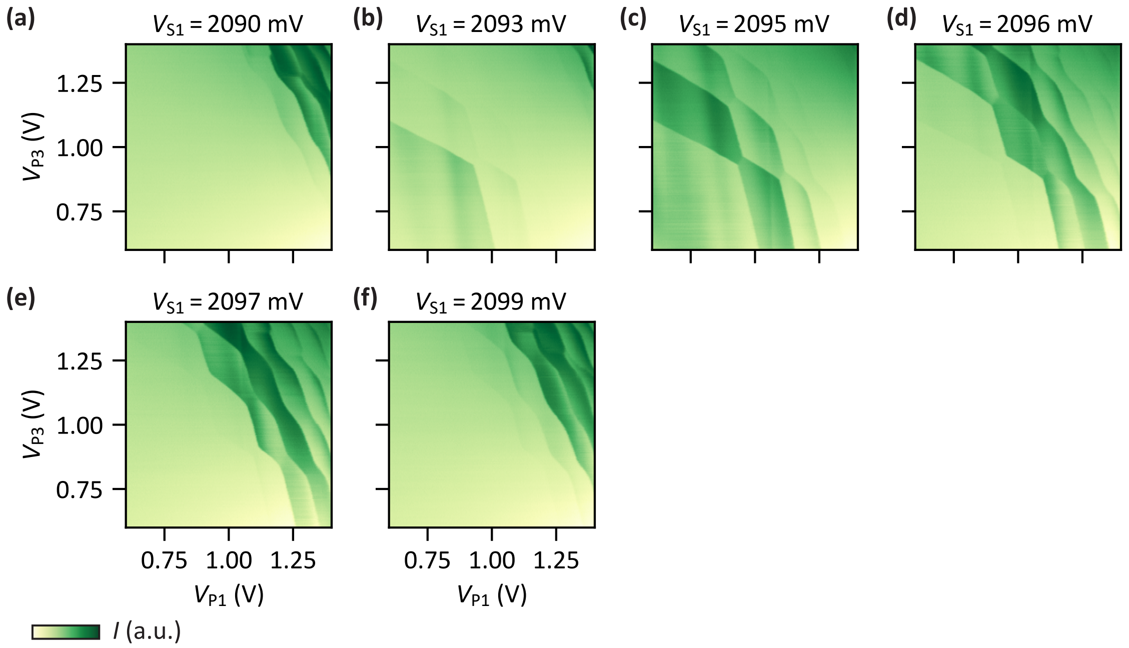

Figure S10. **Charge stability diagrams underlying Fig. 5.a of the main text. (a)-(f)** Multiple charge stability diagrams showing charge transition lines of quantum dot Q1 and Q3. Maps are taken at various sensor gate S1 voltages as indicated above the plots.

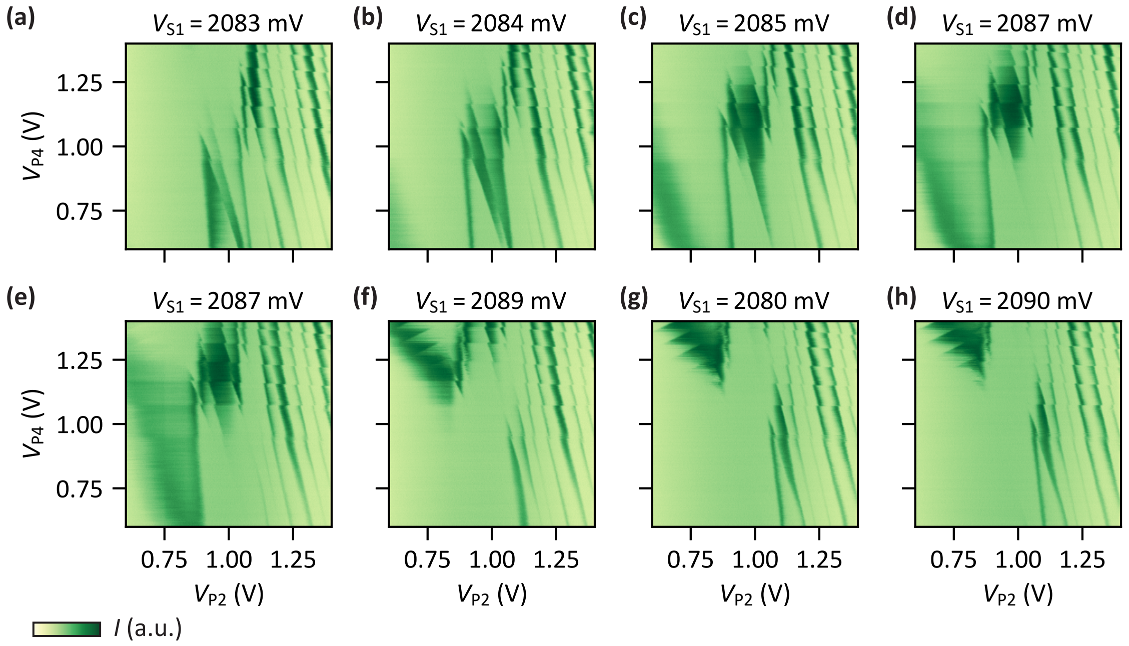

Figure S11. **Charge stability diagrams underlying Fig. 5.b of the main text. (a)-(e)** Multiple charge stability diagrams showing charge transition lines of quantum dot Q1-4. Maps are taken at various sensor gate S1 voltages as indicated above the plots.

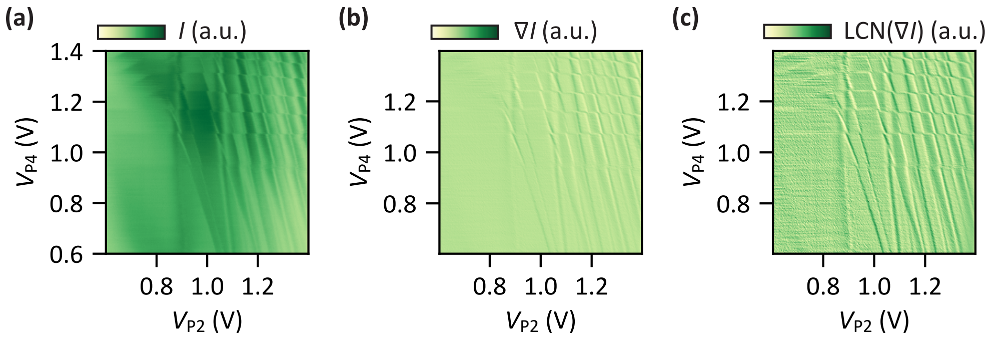

Figure S12. **Processing of the data underlying Fig. 5.b of the main text.** **(a)** Sum of the sensor response I of the charge stability diagrams shown in Fig. S11. **(b)** Gradient ∇I of the data shown in **(a)**. **(c)** Final signal LCN(∇I) after applying a local contrast normalization to the map shown in **(b)**.

S10. OVERVIEW OF APPLIED GATE VOLTAGE CONFIGURATIONS

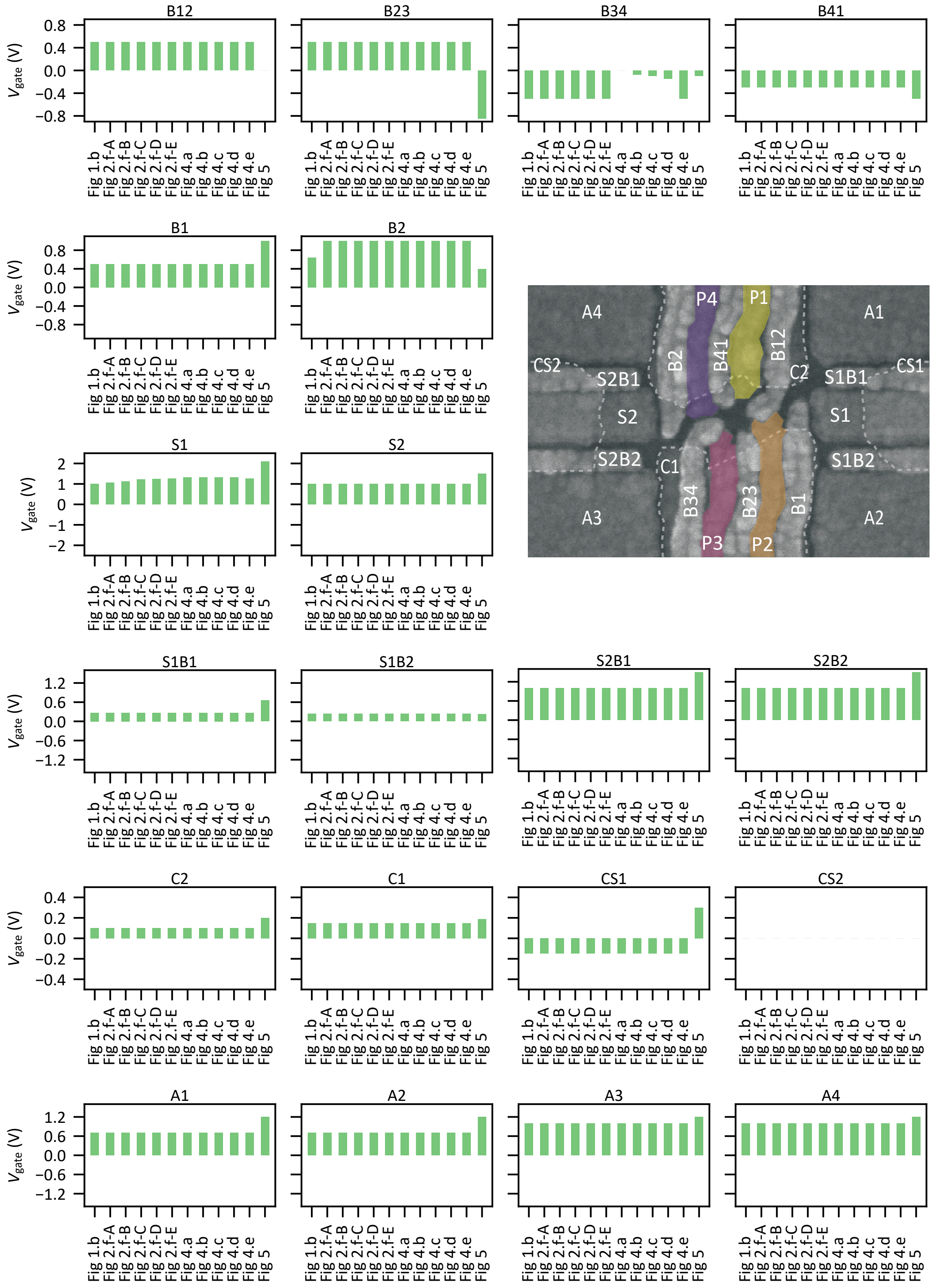

Figure S13. **Gate voltage evolution during the presented experiments.** Each panel shows the gate voltage evolution of a single gate during the experiments presented in the figures of the main text as given on the x-axis. Note that $V_{S2C} = 0$ V during all experiments. The inset shows an SEM image of a device nominally identical to the one under study. Confinement gates are outlined by a white dashed line. Labels indicate the gate electrode naming convention utilized throughout the manuscript and in the panels of this figure.

REFERENCES

[1] D. Degli Esposti, L. E. A. Stehouwer, O. Gül, N. Samkharadze, C. Déprez, M. Meyer, I. Meijer, L. Tryputen, S. Karwal, M. Botifoll, J. Arbiol, S. V. Amitonov, L. M. K. Vandersypen, A. Sammak, M. Veldhorst, and G. Scappucci, Low disorder and high valley splitting in silicon, arXiv [cond-mat.mes-hall] (2023), arxiv.org/abs/2309.02832v3 (accessed 2023-11-29).

[2] W. I. L. Lawrie, H. G. J. Eenink, N. W. Hendrickx, J. M. Boter, L. Petit, S. V. Amitonov, M. Lodari, B. Paquelet Wuetz, C. Volk, S. G. J. Philips, G. Droulers, N. Kalhor, F. van Riggelen, D. Brousse, A. Sammak, L. M. K. Vandersypen, G. Scappucci, and M. Veldhorst, Quantum dot arrays in silicon and germanium, Applied Physics Letters **116**, 080501 (2020).

[3] C. H. Yang, A. Rossi, N. S. Lai, R. Leon, W. H. Lim, and A. S. Dzurak, Charge state hysteresis in semiconductor quantum dots, Applied Physics Letters **105**, 18 (2014).

[4] T. M. Lu, C.-H. Lee, S.-H. Huang, D. C. Tsui, and C. W. Liu, Upper limit of two-dimensional electron density in enhancement-mode Si/SiGe heterostructure field-effect transistors, Applied Physics Letters **99**, 153510 (2011).

[5] C.-T. Huang, J.-Y. Li, K. S. Chou, and J. C. Sturm, Screening of remote charge scattering sites from the oxide/silicon interface of strained Si two-dimensional electron gases by an intermediate tunable shielding electron layer, Applied Physics Letters **104**, 243510 (2014).

[6] D. Laroche, S. H. Huang, E. Nielsen, Y. Chuang, J. Y. Li, C. W. Liu, and T. M. Lu, Scattering mechanisms in shallow undoped Si/SiGe quantum wells, AIP Advances **5**, 107106 (2015).

[7] K.-Y. Chou, N.-W. Hsu, Y.-H. Su, C.-T. Chou, P.-Y. Chiu, Y. Chuang, and J.-Y. Li, Temperature dependence of DC transport characteristics for a two-dimensional electron gas in an undoped Si/SiGe heterostructure, Applied Physics Letters **112**, 083502 (2018).

[8] Y.-H. Su, K.-Y. Chou, Y. Chuang, T.-M. Lu, and J.-Y. Li, Electron mobility enhancement in an undoped Si/SiGe heterostructure by remote carrier screening, Journal of Applied Physics **125**, 235705 (2019).

[9] E. J. Connors, J. J. Nelson, H. Qiao, L. F. Edge, and J. M. Nichol, Low-frequency charge noise in Si/SiGe quantum dots, Physical Review B **100**, 165305 (2019).

[10] T. Struck, A. Hollmann, F. Schauer, O. Fedorets, A. Schmidbauer, . Sawano, H. Riemann, N. V. Abrosimov, L. Cywinski, D. Bougeard, and L. R. Schreiber, Low-frequency spin qubit energy splitting noise in highly purified ^{28}Si/SiGe, npj Quantum Information **6**, 40 (2020).

[11] E. J. Connors, J. Nelson, L. F. Edge, and J. M. Nichol, Charge-noise spectroscopy of Si/SiGe quantum dots via dynamically-decoupled exchange oscillations, Nature Communications **13**, 940 (2022).

[12] A. Goetzberger, V. Heine, and E. H. Nicollian, Surface states in silicon from charge in the oxide coating, Applied Physics Letters **12**, 95 (1968).

[13] E. H. Poindexter and P. J. Caplan, Electron spin resonance of inherent and process induced defects near the Si/SiO_2 interface of oxidized silicon wafers, Journal of Vacuum Science & Technology A **6**, 1352 (1988).

[14] P. M. Lenahan and J. F. Conley Jr., What can electron paramagnetic resonance tell us about the Si/SiO_2 system?, Journal of Vacuum Science & Technology B: Microelectronics and Nanometer Structures Processing, Measurement, and Phenomena **16**, 2134 (1998).

[15] A. Stesmans, T. Nguyen Hoang, and V. V. Afanas'ev, Hydrogen interaction kinetics of Ge dangling bonds at the $Si_{0.25}Ge_{0.75}/SiO_2$ interface, Journal of Applied Physics **116**, 044501 (2014).

[16] T. Thorbeck and N. M. Zimmerman, Formation of strain-induced quantum dots in gated semiconductor nanostructures, AIP Advances **5**, 087107 (2015).

[17] R. M. Stein, Z. S. Barcikowski, S. J. Pookpanratana, J. M. Pomeroy, and J. Stewart, M. D., Alternatives to aluminum gates for silicon quantum devices: Defects and strain, Journal of Applied Physics **130**, 115102 (2021).

[18] K. Vanheusden, W. L. Warren, D. M. Fleetwood, J. R. Schwank, M. R. Shaneyfelt, B. L. Draper, P. S. Winokur, R. A. B. Devine, L. B. Archer, G. A. Brown, and R. M. Wallace, Chemical kinetics of mobile-proton generation and annihilation in SiO_2 thin films, Applied Physics Letters **73**, 674 (1998).

[19] Y.-H. Su, Y. Chuang, C.-Y. Liu, J.-Y. Li, and T.-M. Lu, Effects of surface tunneling of two-dimensional hole gases in undoped Ge/GeSi heterostructures, Physical Review Materials **1**, 044601 (2017).

[20] M. Meyer, C. Déprez, T. van Abswoude, I. Meijer, D. Liu, C.-A. Wang, S. Karwal, S. Oosterhout, F. Borsoi, A. Sammak, N. W. Hendrickx, G. Scappucci, and M. Veldhorst, Electrical control of uniformity in quantum dot devices, Nano Letters **23**, 2522 (2023).
